# Genetic and epigenetic alterations in the *GNAS* locus and clinical consequences in Pseudohypoparathyroidism: Italian common healthcare pathways adoption

**DOI:** 10.1186/s13052-016-0310-3

**Published:** 2016-11-21

**Authors:** L. de Sanctis, F. Giachero, G. Mantovani, G. Weber, M. Salerno, G. I. Baroncelli, M. F. Elli, P. Matarazzo, M. Wasniewska, L. Mazzanti, G. Scirè, D. Tessaris, On behalf of the Study Group Endocrine diseases due to altered function of Gsα protein of the Italian Society of Pediatric Endocrinology and Diabetology (ISPED)

**Affiliations:** 1 Department of Public Health and Pediatric Sciences, University of Turin - Regina Margherita Children’s Hospital – Health and Science City, Subintensiva Allargata Prima Infanzia, Piazza Polonia 94, 10126 Torino, Italy; 2 Kinderklinik, Evangelisches Krankenhaus Oberhausen, Oberhausen, Germany; 3 Fondazione IRCCS Ca’ Granda Ospedale Maggiore Policlinico, Endocrinology Unit, Department of Clinical Sciences and Community Health, University of Milan, Milan, Italy; 4 Department of Pediatrics, San Raffaele Hospital, University of Milan, Milan, Italy; 5 Pediatric Endocrine Unit, Department of Translational Medical Sciences, University of Naples Federico II, Naples, Italy; 6 Department of Obstetrics, Gynecology and Pediatrics, I Pediatric Division, University Hospital, Pisa, Italy; 7 Pediatric Endocrinology and Diabetology Unit, Regina Margherita Children’s Hospital – Health and Science City, Turin, Italy; 8 Department of Pediatric, Gynecological, Microbiological and Biomedical Sciences, University of Messina, Messina, Italy; 9 Pediatric Endocrinology and Rare Diseases, Department of Pediatrics, S.Orsola-Malpighi Hospital, University of Bologna, Bologna, Italy; 10 Endocrinology Ward, Bambin Gesù Children’s Hospital, Rome, Italy

**Keywords:** *GNAS* gene, *GNAS* locus, Pseudohypoparathyroidism, Albright Hereditary Osteodystrophy, PTH resistance

## Abstract

**Background:**

Genetic and epigenetic alterations in the *GNAS* locus are responsible for the Gsα protein dysfunctions causing Pseudohypoparathyroidism (PHP) type Ia/c and Ib, respectively. For these heterogeneous diseases characterized by multiple hormone resistances and Albright’s Hereditary Osteodystrophy (AHO) the current classification results inadequate because of the clinical overlap between molecular subtypes and a standard clinical approach is still missing.

In the present paper several members of the Study Group Endocrine diseases due to altered function of Gsα protein of the Italian Society of Pediatric Endocrinology and Diabetology (ISPED) have reviewed and updated the clinical-molecular data of the largest case series of (epi)/genetically characterized AHO/PHP patients; they then produced a common healthcare pathway for patients with these disorders.

**Methods:**

The molecular analysis of the *GNAS* gene and locus identified the causal alteration in 74 subjects (46 genetic and 28 epigenetic mutations). The clinical data at the diagnosis and their evolution during up to 15 years follow-up were collected using two different cards.

**Results:**

In patients with genetic mutations the growth impairment worsen during the time, while obesity prevalence decreases; subcutaneous ossifications seem specific for this group. Brachydactyly has been detected in half of the subjects with epigenetic alterations, in which the disease overts later in life, often with symptomatic hypocalcaemia, and also early TSH and GHRH resistances have been recorded.

**Conclusions:**

A dedicated healthcare pathway addressing all these aspects in a systematic way would improve the clinical management, allowing an earlier recognition of some PHP features, the optimization of their medical treatment and a better clinical-oriented molecular analysis. Furthermore, standardized follow-up data would provide new insight into less known aspects.

## Background

Pseudohypoparathyroidism (PHP) defines a group of rare heterogeneous metabolic disorders, characterized by resistance to the peripheral action of PTH, the most important hormone regulating the calcium and phosphorus homeostasis [1, 2]. Several mutations of the *GNAS* gene (20q13.32) and epigenetic alterations within its locus have been described as causes of the different forms of PHP type I (i.e. PHP-Ia, PHP-Ib, PHP-Ic). They all induce an impaired function of the Gsα protein (the α-subunit of the heterotrimeric stimulatory G protein), which regulates the adenylate cyclase activity in the signaling pathway of various peptide hormones binding the G-protein-coupled receptors (GPCR): PTH, TSH, GHRH, gonadotropins, ACTH, and calcitonin among others [1, 3].

Inactivating mutations in exons 1–13 of the maternal copy of the *GNAS* gene lead to PHP-Ia, whose phenotype encompasses the Albright Hereditary Osteodystrophy (AHO) signs including short stature (SS), brachydactyly (BR), obesity (OB), round face (RF), subcutaneous ossifications (SO) and mental retardation (MR) together with multiple resistances to PTH (rPTH), TSH (rTSH) and to other aforementioned GPCR-binding hormones. Altered response to other hormones (i.e. insulin) were recently debated as part of the phenotype [4, 5]. In several subjects with the same clinical features but normal Gsα protein in vitro activity (i.e. PHP-Ic patients) mutations affecting the C-term region of the *GNAS* gene and its binding site to the GPCRs were described [6, 7]. A different form of the disease, named Pseudopseudohypoparathyroidism (PPHP) and characterized by isolated AHO features without hormone resistances, occurs if germline mutations involve the paternal copy of the gene [8]. PHP-Ia and PPHP may affect members of the same family and such different clinical presentation have been explained by tissue-specific differential methylation patterns [9].

The PHP-Ib subgroup is classically defined by rPTH, without AHO signs nor other hormone disruptions, except at times rTSH [1]; and it is caused by epigenetic alterations at the *GNAS* locus, that is one of the most complex ones in the human genome. In addition to Gsα, it gives rise to four other transcripts (XLαs, A/B, NESP55 and AS) [10] whose expression is regulated by an imprinting mechanism [11]. The loss of methylation on maternal exon A/B is the most common alteration: in the familial autosomal dominant form of the disease (i.e. AD-PHP-Ib) this involves deletions in the *STX16* gene, although in some families it was also related to NESP55 and NESP-AS alterations [12, 13]. Conversely, the sporadic form of PHP-Ib shows multiple alterations of the imprinting pattern (loss of methylation at NESP-AS, XLαs, A/B and gain of methylation at NESP55); paternal isodisomy of chromosome 20q involving the *GNAS* locus has also been described in some patients with large imprinting disruptions [14, 15].

In the last years several independent studies have reported complex clinical phenotype encompassing AHO features and multiple hormone resistances in patients with molecular diagnosis of PHP-Ib, suggesting that altered methylation may play a similar role to structural mutations in the pathogenesis of the disease [16–19]. Conversely, in subjects with AHO and multihormone resistances, with normal Gsα protein in vitro activity, clinically indicated as having the PHP-Ic subtype, the same alterations found in the *GNAS* locus have recently been described [19]. Furthermore, *GNAS* gene mutations were described also in patients with Progressive Osseous Heteroplasia (POH) and primary Osteoma Cutis (OC) broadening the spectrum of Gsα-related disorders and representing two important differential diagnosis [20, 21]. Thus the clinical overlap between different genotypes made the current classification inadequate pointing out the need of an update.

On the other hand current literature data show a lack of knowledge about some clinical features [22]: prevalence of several clinical signs are often debated, and to date only few studies have considered the evolution of the disease over the time. Likewise, due to the rareness of the disease, a common clinical approach is still missing and no standards or recommendations are available for the clinical routine.

In the largest series of PHP patients with a confirmed molecular diagnosis this work attempts to describe the genotype-phenotype correlation and to give a first insight into the evolution of the disease in a mainly pediatric population. To improve the standardization of the clinical management and further data collection of PHP patients we also introduce a possible flowchart for a common healthcare pathway.

## Methods

### Patients

Since 1999, patients with clinical diagnosis of PHP, defined as rPTH (i.e. raised serum PTH levels, in presence of hyperphosphatemia and normo- or hypocalcaemia, despite normal renal function [16]) isolated or associated to rTSH or AHO signs, were collected countrywide by the main Italian referee Centers for Pediatric Endocrinology within the research project *Molecular analysis of the GNAS gene in subjects with suspected PHP* of the Study Group *Endocrine diseases due to altered function of Gsα protein* of the Italian Society of Pediatric Endocrinology and Diabetology (ISPED). DNA samples and clinical data were sent to the Department of Public Health and Pediatrics of the University of Turin, where the molecular analyses were performed by searching for mutations within the coding region and intron-exon boundaries of the *GNAS* gene.

In patients with wild-type *GNAS* sequence, the cooperation of the Endocrinology Unit at Fondazione IRCCS Ca’ Granda Policlinico - University of Milan allowed to expand the molecular diagnosis by further methylation analysis on the *GNAS* locus.

The causal alteration was detected in a total of 74 subjects, from 69 unrelated families. The principal clinical features and the molecular characterization of the case series are presented in Tables 1, 2 and 3.

**Table 1:** Principal features at diagnosis in the 2 groups of patients with *GNAS* gene mutations and *GNAS* locus altered methylation

	N° Subjects	Sex: F/M	Age	Ca (mEq/l)	P (mg/dl)	PTH (pg/ml)	TSH (μU/ml)
Gene mutation	46	29/17	4.8 (0.1–23.4)	4.3 (2.25–6.7)	6.8 (4–12.2)	308 (15.6–1200)	7.8 (1.6–22.1)
Locus altered methylation	28	16/12	10.5 (0.5–65)	3.6 (1.9–9.2)	6.9 (3.6–10.5)	358 (128–1532)	5 (3.36–8.95)

Legend. Sex: Females/Males; Age: median age in years (range); Ca: median total serum Calcium levels (range); P: median serum Phosphate level (range); PTH: median serum PTH (range); TSH: median serum TSH level (range)

**Table 2:** Clinical features and molecular characterization of patients with *GNAS gene* mutations

Pt	Sex	Age	AHO signs	Hormone resistances	GNAS gene mutation
A1	F	0.25	OB	rPTH, rTSH	c.240 + 1G > T
A2	F	2	BR, SO, MR, (OB), RF	rPTH, **rTSH (12)**	c.347_348insC
A3	M	3.68	BR, MR, OB, RF	rPTH, rTSH	c.347_348insT
A4	F	1.53	BR, SS, (OB), RF	rPTH, rTSH	c.1009C > T
A5	F	3.31	BR*, MR, OB, RF	rPTH, rTSH	c.421_422del
A6	M	4.72	BR, SO, MR, (OB/OW), RF	rPTH, rTSH	c.112delC
A7	F	5.8	BR*, **SS (6.8),** OB/OW, RF	rPTH, rTSH	c.1177G > T
A8	F	7.19	BR, **SO (13), OB/OW (10.7)**, RF	rPTH, rTSH	c.348C > T
A9	M	23.17	BR, MR, SS	rTSH, rFSH/LH	c.478G > A
A10	F	0.95	SO, OB, RF	rPTH, rTSH	c.1009C > T
A11	M	8.2	BR, SO, MR, SS, RF	rPTH, rTSH, rFSH/LH	c.103C > T
A12	F	12.73	BR, OB/OW, RF	rPTH, rTSH	c.1177G > T
A13	M	12.72	SS, RF	rPTH, rTSH	c.1177G > T
A14	F	4.45	BR, SO, MR, RF	rPTH, rTSH, rFSH/LH	c.523_524del
A15	M	4.45	BR, SO, MR, SS, OB/OW, RF	rPTH, rTSH	c.523_524del
A16	M	6.2	SO, MR	rPTH, rTSH	c.212 + 2_212 + 6del
A17	F	2	**BR (10.3),** SO, (OB)	rPTH, rTSH, **rGHRH (10.3)**	c.103C > T
A18	F	23.41	BR, SO, MR, OB, RF	rPTH, rTSH	c.103C > T
A19	F	7.2	BR	rPTH, rTSH	c.742G > C
A20	F	10.47	BR, OB/OW	rPTH, rTSH	c.742G > C
A21	M	11.01	BR, SS, OB, RF	rPTH, rTSH, rLH/FSH	c.1009C > T
A22	M	2	BR* (8), SO, MR**,** OB (0.5), **RF(11)**	rTSH, **rPTH (8)**	c.521_522del
A23	M	11.23	BR, MR, OB/OW, RF	rPTH, rTSH	c.805A > G
A24	M	10.1	BR, SO, MR, RF	rPTH, rTSH	c.568_571del
A25	F	9.75	BR, MR, SS, OB/OW, RF	rPTH, rTSH, rFSH/LH rIns	c.91C > T
A26	F	1.5	BR, SO, MR, OB, RF	rPTH, rTSH, rIns	c.91C > T
A27	F	1.5	SO, RF	rPTH, rTSH	c.568_571del
A28	F	4.6	BR, SO, MR, SS, OB/OW, RF	rPTH, rTSH	c.568_571del
A29	M	1.3	MR, OB, RF	rPTH, rTSH	c.568_571del
A30	M	1.3	BR, **MR (2.8)**, SS, (OB), RF	**rPTH (2.8)**, rTSH, rGHRH	c.21dupT
A31	F	1.54	SO, MR, OB/OW, RF	**rTSH (1.9), rPTH (4)**	c.347_348insT
A32	F	8.86	BR, MR, OB, RF	rPTH, rTSH	c.481C > T
A33	F	8.43	BR, MR, RF	rPTH, rTSH	c.568_571del
A34	F	14	BR, MR, SS, OB/OW, RF	rPTH, rTSH	c.728C > T
A35	M	12.77	BR, SO	rPTH, rTSH	c.110del
A36	F	16.8	BR, SO, MR, OB/OW, RF	rPTH, rTSH	c.1021_1022ins23nt
A37	F	4.89	BR*, MR, OB, RF	rPTH, rTSH	c.863_864del
A38	F	3.56	BR, SO, SS, OB/OW, FR	rPTH, rTSH	c.87dupA
A39	F	0.1	BR, SO, MR, (OB 0.1), RF	rPTH, rTSH,	c.568_571del
A40	F	0.94	BR*, SS, (OB/OW), RF	rPTH	c.1177G > A
A41	M	2.32	BR, SO, OB/OW, RF	rPTH,	c.347_348insC
A42	F	13.86	BR, MR, RF	rTSH	c.97G > A
A43	F	0.91	BR, MR, SS, OB, RF	rPTH, rTSH	c.363_364del
A44	F	14.38	BR, SS	--	c.568_571del
A45	M	14.15	BR, SO	rPTH	c.103C > T
A46	M	6.61	SO, **OB/OW (9.11)**	rPTH	c.103C > T

Features that occurred prior to the first diagnosis or later in life are remarked in bulk and the corresponding age is noticed in parenthesis. Features that were present at the diagnosis but disappeared later in life are reported in parenthesis. A12 + A13; A14 + A15; A19 + A20; A25 + A26; A45 + A46 are couple of brothers and/or sisters

**Table 3:** Clinical features and molecular characterization of patients with *GNAS locus* altered methylation

Case	Sex	Age	AHO signs	Hormone resistances	*GNAS* locus methylation alteration
B1	M	6.9	BR, MR, RF	rPTH, **rTSH (11)**	AB/NESP/AS/XL
B2	M	11.43	**MR (3.5)**, RF	rPTH	AB/NESP/AS/XL
B3	F	7.5	--	rPTH	AB/NESP/AS/XL
B4	F	65	BR, OB, RF	rPTH, rLH/FSH	AB/NESP/AS/XL
B5	F	5.2	MR, OB/OW, RF	rPTH, rTSH	AB/NESP/AS/XL
B6	F	9.95	RF	rPTH	AB/NESP/AS/XL
B7	M	15	BR	rPTH	AB/NESP/AS/XL
B8	F	13.26	--	rPTH	AB/NESP/AS/XL
B9	M	12.13	--	rPTH	AB/NESP/AS/XL
B10	F	6.55	MR, OB/OW	rPTH, rTSH	AB/NESP/AS/XL
B11	M	10.5	BR, OB/OW, RF	rPTH	AB/NESP/AS/XL
B12	M	37.5	BR, SS,OB/OW, RF	rPTH	AB/NESP/AS/XL
B13	F	9	RF	rPTH	AB/NESP/AS/XL
B14	F	0.5	OB/OW, RF	**rPTH,** rTSH	AB/NESP/AS/XL
B15	M	12.76	RF	rPTH	AB/NESP/AS/XL
B16	M	12.23	**BR**	rPTH	AB/NESP/AS/XL
B17	M	12	BR, OB, RF	rPTH, rTSH	AB/NESP/AS/XL
B18	M	16	BR*, OB/OW, RF	rPTH, **rGHRH**	AB/NESP/AS/XL
B19	F	4	BR*, **OB/OW,** RF	rPTH, rTSH	AB/NESP/AS/XL
B20	F	4.16	MR, OB/OW, RF	rPTH, rTSH	AB/NESP/AS/XL
B21	F	10.09	RF	rPTH, rTSH	AB/NESP/AS/XL
B22	F	7,41	BR	rPTH	AB/NESP/AS/XL
B23	F	5.5	BR, MR, RF	rPTH	AB/NESP/AS/XL
B24	F	23.5	BR, MR	rPTH	AB/NESP/AS/XL
B25	F	3.06	**BR**	rPTH, **rTSH (6)**	AB/NESP/AS/XL
B26	M	29	BR*, OB/OW	rPTH	Del at *STX16* gene
B27	M	12.9	--	rPTH	Del at *STX16* gene
B28	F	19.6	SS, OB/OW, RF	rPTH	Del at *STX16* gene

Sex: Female (F), Male (M); Age: age at diagnosis or at first evaluation, expressed in years; AHO signs: brachydactyly (BR, in case of X-Ray study: BR*), subcutaneous ossifications (SO), mental retardation (MR), short stature (SS), obesity and overweight (OB/OW), round face (RF); Hormone resistances: PTH resistance (rPTH), TSH resistance (rTSH), FSH/LH resistance (rFSH/LH), GHRH resistance (rGHRH), Insulin resistance (rIns). Features that occurred prior to the first diagnosis or later in life are remarked in bulk and the corresponding age is noticed in parenthesis. Features that were present at the diagnosis but disappeared later in life are reported in parenthesis. A12 + A13; A14 + A15; A19 + A20; A25 + A26; A45 + A46 are couple of brothers and/or sisters

Structural mutations in exons 1–13 were detected in 42 subjects (4 couples of siblings), thus diagnosed as PHP-Ia; in 4 patients (A17, A18, A45 and A46) the phenotype was characterized by early and widespread subcutaneous ossifications, raising a differential diagnosis with POH. Four other patients (A7, A12, A13 and A40). 2 of them siblings, harbored the mutation affecting the C-terminal region of the Gsα protein (exon 13), associated to normal in vitro Gsa activity [6], allowing the definition of the more rarely described PHP-Ic. The pathogenicity of each variant was checked within the 1000 genomes database (www.internationalgenome.org) and the mutation nomenclature within the Leiden Open Variation Database (www.lovd.nl/3.0).

All 28 patients with epigenetic alterations in the *GNAS* locus came from unrelated families. Altered methylation patterns in multiple DMRs were detected in most of them (*n* = 25, B1-25), allowing the diagnosis of sporadic PHP-Ib. In the 3 remaining patients (B26-28) a deletion on the *STX16* gene responsible for the familial form of the disease (AD-PHP-Ib) has been identified.

Clinical data at the time of the enrollment and during the follow-up period were collected through 2 different recording cards. The first one has been conceived to assess the typical PHP signs, leading to the clinical suspicion of the disease, i.e. the presence/absence of the AHO signs and of PTH and TSH resistances; subsequently, through a new research project, named *Clinical Update about patients with PHP/AHO* a new follow-up card has been drawn to collect further clinical details about patients with a confirmed molecular diagnosis. In particular it has been focused on the evolution of auxological parameters, on the possible late onset of some AHO signs and other hormone resistances, overall the less known ones. This second part of the study is still going on in a clinical network with the aim of taking care of the patients during the whole pediatric age, and when possible also later in life (through cooperation with adult age endocrinologists).

Informed consent was obtained for all patients involved in the study from either parents (or legal guardian), or from the patients themselves in case of adults.

In the 2 cards, in addition to rPTH and the onset age, rTSH was considered in presence of clinical or subclinical hypothyroidism (i.e. high serum TSH, with or without low serum level of free Thyroxine); other hormone defects (resistances to GHRH (rGHRH), FSH/LH (rGn) and reduced insulin sensitivity) were identified by hormone increased basal levels or altered response to the specific stimulation tests.

SS was diagnosed as height below the 3rd percentile for chronological age, while OB was defined as Body Mass Index (BMI) above the 97th percentile in children (SDS ≥ 1.88) and above 30 kg/m^2^ in adults; overweight (BMI ≥ 85th percentile or SDS ≥ 1.036 in children and BMI ≥ 25 kg/m2 in adults) was also marked but not considered for clinical diagnosis of PHP. The aforementioned auxological parameters have been evaluated through the software *Growth Calculator 3.0* (available on the ISPED’s web site http://www.siedp.it/pagina/151/growth+calculator+3) that gives access to several growthcharts designed on different populations. Since our patients had Italian origin, we employed the Italian charts (INeS’ Charts and Cacciari’s Charts [23]) in order to obtain uniformity.

BR was recorded if clinically present with positive Archibald’s sign (in case of shortening of IV-V metacarpals) and/or shown by the Metacarpophalangeal profile (MPP, indicating at least one metacarpal bone or distal phalanx shorter than 2 SD score – SDS) [16]. SO were reported if clinically evident or confirmed by X-rays; in few patients bioptic analysis has been performed before surgical removal.

RF was reported as clinical feature, while MR was marked in case of psychomotor retardation or delayed speech or need of assistant teacher and extra school help.

### Molecular analysis

#### GNAS gene analysis

Genomic DNA was extracted from peripheral blood leukocytes using the commercial kit PUREGENE® DNA Purification Kit, Gentra. The 13 coding exons of the *GNAS* gene, together with the exon-intron boundaries regions were amplified by PCR using the ABI PRISM® BigDye Terminator Cycle Sequencing Kit and then directly sequenceed by using the ABI PRISM® Genetic Analyzer 3100 (Applied Biosystems, Foster City, CA). Each mutation has been searched for and excluded in 50 control subjects as previously described [16]. For few mutations in vitro mutant expression has been tested [6].

#### Methylation analysis of GNAS locus and detection of STX deletions

*GNAS* DMRs methylation status was assessed both by combined bisulfite restriction analysis and methylation specific-multiplex ligand-dependent probe amplification (MS-MLPA). The presence of STX16 gene deletions was investigated by multiplex PCR and MS-MLPA, as previously described [8, 16, 24].

### Statistical analysis

To explore the relationship among clinical features and molecular diagnosis we used the *Mann–Whitney U* and *Fisher* nonparametric tests, available as open access tools on the *VassarStats: Website for Statistical Computation* (http://vassarstats.net). A two-tailed *P* value of <0.05 was considered statistically significant and results are presented as median value and range.

## Results

The two groups of patients differ in the age of onset of the disease (*p* < 0.001): patients with genetic mutations came to medical attention at a median age of 4.8 years (range 0.1–23.4) and in 29/46 cases (63%) the reasons for consultation were AHO signs (early-onset obesity, delayed growth, heterotopic ossifications) and/or hormonal alterations (overall hypothyroidism); 4/46 patients only showed symptomatic hypocalcaemia as first sign. Conversely, patients with locus alterations were older at diagnosis (median age 10.5 years; range 0.5–65), most of them (*n* = 20/28, 70%) presenting with seizures, tetany, and positive Trousseau’s sign.

**Fig. 1:**
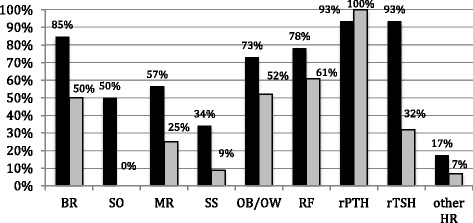
Prevalence of AHO signs and hormone resistances in the 2 group of patients with *GNAS* gene mutations (*dark grey bars*) and *GNAS* locus mutations (*light grey bars*): encompassing brachydactyly (BR) (*p*- value 0.002), subcutaneous ossifications (SO), mental retardation (MR) (p value 0.008), short stature (SS) (*p* value 0.04), obesity and overweight (OB/OW) (*p* value 0.16), round face (RF) (*p* value 0.18), PTH resistance (rPTH), TSH resistance (rTSH) (*p* value <0.0001), other hormone resistances (HR) (*p* value 0.37)

Clinical data prevalence in the studied series is resumed in Fig. 1: PTH resistance was detected in most patients with genetic mutations (*n* = 43/46, 93%) at a median age of 5 years (range 0.9–23.4), while all patients with imprinting alterations showed rPTH at diagnosis, often occurring later in life (median age 11.6 years; range 0.5– 65), with symptomatic hypocalcaemia. TSH resistance arose in 43/46 patients (93%) with genetic mutations at median age of 5.8 years (range 0.25–23.4) and in 9/28 patients (32%) with imprinting alterations at median age of 6.28 years (range 0.5–11).

Other hormone resistances were reported in 8/46 patients with genetic mutations (17%) only, but notably 44 of the total case series were younger than 12 years at the time of last evaluation. Gonadotropins resistance was recorded in 5 patients, associated to insulin resistance in one of them (A26), which is the further isolated hormonal resistance in her sister (A25). GH defect was detected at diagnosis in 2 patients: A30 (1.3 and 5 years old at diagnosis and follow-up, respectively) showed short stature and a lack of response to the GHRH + Arginine test but a second test was not performed because his height velocity (HV) has improved; and A17 (2 and 10.3 years old at diagnosis and follow-up, respectively), displayed GH deficiency after two stimulation tests. Therefore she underwent a treatment with hrGH with HV improvement and normalization of the height to target height.

Among patients with epimutations, in one patient (B18) only GHRH resistance has been reported, occurring in adult age; for a female patient (B4) hypofertility was referred when she was 29, but she came late to medical observation with incidental diagnosis of hypocalcaemia as possible sign of mild hormonal alteration.

**Fig. 2:**
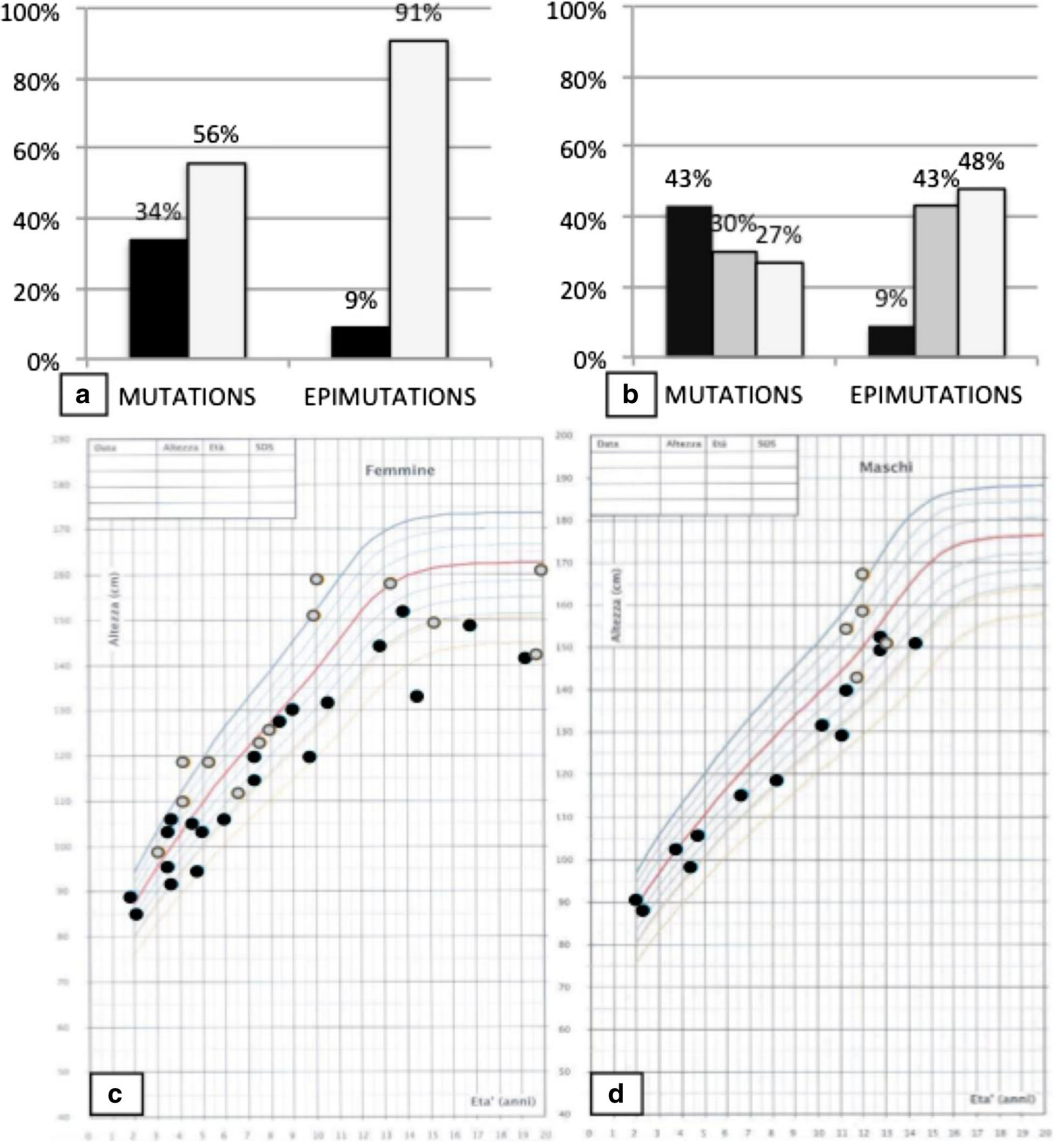
**a** Prevalence of short (*black bars*) compared to normal stature (*white bars*), between the 2 groups of patients with *GNAS* gene mutations (named “Mutations”) and *GNAS* locus imprinting alterations (named “Epimutations”). **b** Prevalence of weight excess (overt obesity: *black*; overweight: *grey*) compared to normal weight (*white*) at the time of enrollment between two groups with *GNAS* gene mutations (named “Mutations”) anf *GNAS* locus imprinting alterations (named “Epimutations”). **c** and **d**: Heights of the presented case series at the time of enrollment on females (section **c**) and males growth chart (section **d**); patients with *GNAS* gene mutations are presented by *blue plots* and those with *GNAS* locus imprinting alterations by *yellow plots*

Auxological evaluations were obtained (Fig. 2) in 44/46 patients with genetic mutations and SS became evident in 15 subjects (34%) at a median age of 6.8 years (range 0.91–23.17); 11 of them were still pre-pubertal at the time of the examination (<12 years). Auxological data were available for 23/28 subjects with imprinting alterations and 2 of them (9%) were short at the time of enrollment.

Weight excess was recorded in 32/44 patients with genetic mutations (73%) and it encompassed either early-onset obesity (*n* = 19, 43%) with a median age of 1.5 years (range 0.9–23.4), or overweight (*n* = 13, 30%) later (median age 10.1 years, range 1.5–19.5). Most patients (*n* = 28) with weight excess were pre-pubertal (<12 years) at the time of the evaluation. Only 2/23 subjects with imprinting alterations (9%) showed overt obesity at extremely different ages (1.2 and 65 years); while 10/23 further patients (43%) were overweight (median age 14.4 years, range 4.2–37.5).

**Fig. 3:**
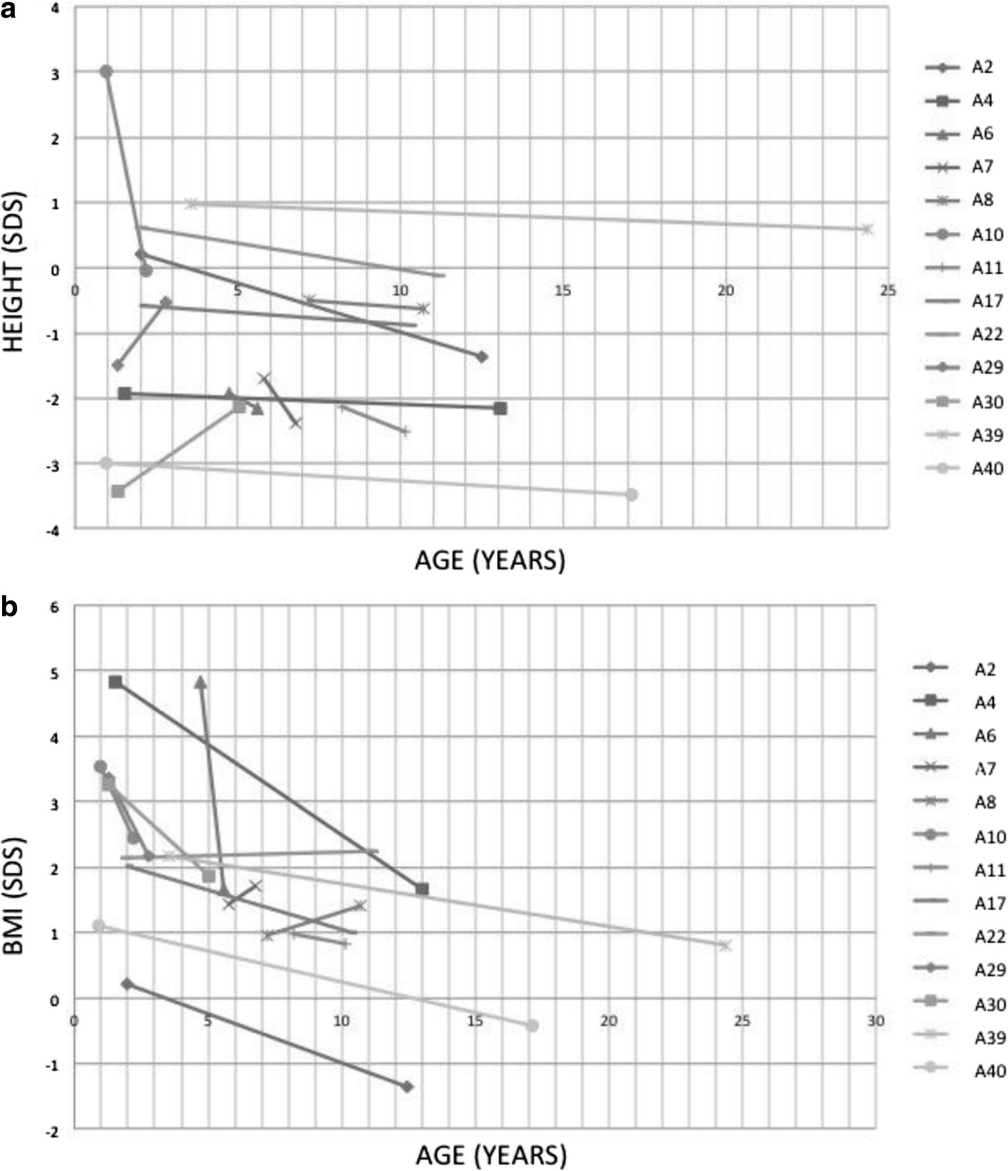
Height (plot **a**) and Weight (plot **b**) auxological data of 13 patients with *GNAS* gene mutations at follow-up: data are presented as Standars Deviation Score (SDS) calculated on age-adapted growth charts vs. age in years (AGES)

Follow-up data in 13 patients with genetic mutations (Fig. 3) demonstrated an evolution of the auxological parameters over the time: during a mean follow-up time of 6.2 years, SS prevalence increased from 31% (median age 1.4 years, range 0.94–8.2) to 38% (median age 10.1 years, range 5.04–17.1), while weight excess prevalence decreased from 77 to 54%. At the time of enrollment 10 very young patients (oldest age 5.8 years) showed weight excess: at follow-up half of them normalized or strongly reduced the weight at a median age of 12.5 years (range 5.6–24.35), while the remaining 5 patients were still obese or overweight (median age 5.0 years, range 2.2–11.2). For all patients, hypocaloric regimens for age and recommendations aimed to increase the physical activity over the time were the only reported therapeutic approaches, without clarification about any further specific treatment.

BR has been identified in 39/46 subjects with genetic mutations (85%) at a median age of 7.2 years (range 0.9–23.4), while the sign was positive in 14/28 subjects with imprinting alterations (50%, *p* = 0.002) and recorded at a later median age of 13.2 years (range 1.2–65; *p* < 0.001). None of the latter showed SO, which were detected in 23/46 subjects harboring genetic mutations (50%), at a median age of 4.5 years (range 1.5–23.4). Interestingly, 9/11 (82%) patients with exon 1 mutations showed SO, which were present in 14/35 (40%) patients only harboring mutations throughout the remaining *GNAS* coding region. Signs of MR were more often observed among mutated than epimutated patients (in 26 and 7 patients respectively, 57 and 25%, *p* = 0.008), but the age at presentation didn’t show any statistical difference (median age was 4.75 years in both groups). Few information were available about the features of the cognitive impairment: among patients with genetic mutations it involved psychomotor (*n* = 4) or speech delay (*n* = 4), school problems with need of extra help (*n* = 2), and a slight global retardation of developmental milestones (*n* = 5); results of objective cognitive tests were available for 5 patients.

**Fig. 4:**
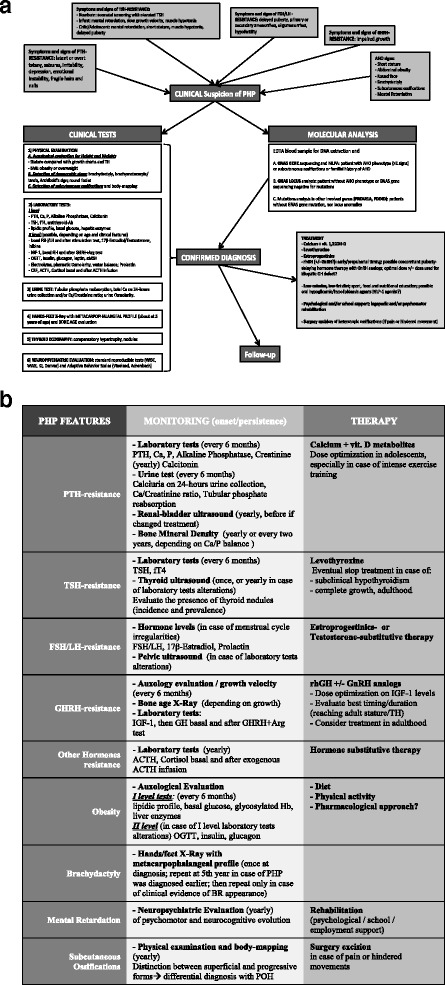
Diagnostic (**a**) and follow-up (**b**) flowcharts for patients with suspected PHP. **a** In the initial diagnostic phase we suggest to assess the presence or absence of each AHO sign and hormone resistance possibly involved in PHP through targeted clinical examinations (physical and neuropsychiatric), laboratory tests (on blood and urine) and medical imaging investigations. This wide diagnostic approach, despite laborious, represents an exhaustive base-line assessment for later comparative examinations. **b** During the follow-up management we propose for each clinical feature involved in PHP a practical checklist of clinical, biochemical and/or imaging investigations to perform periodically or in case of symptoms in the follow-up of PHP patients. In particular, we believe that patients who are very young at diagnosis would benefit of systematic reevaluations, in order to correctly manage late complications of the disease and to detect late-onset signs during the growth for a prompt treatment

The review and update of clinical data of this countrywide collected cohort of patients with confirmed molecular diagnosis of PHP has led us to develop two flow-charts, one for the initial diagnosis and the other for the monitoring of the disease, as common tool for the clinical routine management of these subjects (Fig. 4).

## Discussion

The present study reports the clinical review and update about the largest cohort of patients with a confirmed molecular diagnosis of PHP, in order to propose a common standard of care. Due to the rarity of the disease and to the overlapping phenotype among patients with different genotypes we think that initially it may be more useful to organize the clinical management and follow-up of PHP patients in a unique scheme, independently of their genotype. This approach will possibly help in detecting (and treating) clinical features earlier during the history of the disease. The adoption of a common healthcare pathway will allow the collection of standardized data that may improve the further characterization of the disease, including more precise information about the phenotype/genotype correlation, and the success of treatments.

Similar to previous reports [25, 26] our patients showed a bimodal way of presentation: younger pediatric subjects seeking for medical advice because of early-onset obesity and/or congenital hypothyroidism had more often structural gene mutations, suggesting a more severe impairment of the Gsα protein, not only in endocrine organs as reported by prior functional studies [27], but probably also in adipose tissue. Conversely, symptomatic hypocalcaemia is the most often presentation in peripubertal and older patients harboring locus imprinting alterations: this genotype might induce a slight impairment of the Gsα activity which, thank to a higher production of PTH, would preserve normocalcaemia until puberty or even adulthood, when the increased calcium requirement makes the resistance clinically evident. As regard to rPTH it is nowadays debated how long-term elevated PTH and calcium substitution might interfere on bone health status and BMD [28]: a focused monitoring of calcium/phosphorus metabolism and mineral bone status, also considering the different treatment regimens adopted over the time, might produce more uniform data to gain some new insight into these issues for patients with both genotypes.

Often described as subclinical hypothyroidism [22], rTSH was confirmed as the second most common hormone derangement [29, 30]. Its congenital or early onset in patients with either genetic or epigenetic mutations suggests that it should be searched for at diagnosis in both PHP subtypes; furthermore this finding seems to indicate a greater sensitivity to the Gsα altered pathway in thyroid than in parathyroid cells [26]. In order to define common clinical approaches, standardized follow-up studies are required to assess the optimal TSH-cut-off (TSH 5–10 μU/ml) at which substitutive treatment should be started, its duration until or after the growth has been completed and the timing of TSH levels monitoring.

Although multiple hormone resistances were classically described in PHP-Ia patients [1], their relative low prevalence in our series could in part be explained by the very young age of most subjects, but it also might reflect that neither the historically reported hormonal derangements (involving vasopressin, prolactin, ACTH, CRF, calcitonin and glucagon [31–33]) nor the newly recognized ones (including insulin, leptin and αMSH [5]) are initially investigated and routinely included in the follow-up program. Likewise for rGHRH, reported as a common finding in PHP-Ia subjects, the GH secretory testing should be part of the initial management [22, 34]. A prompt diagnosis of GH deficiency as soon as height velocity decreases, would allow an early replacement treatment overall before the typically observed premature fusion of growth plates hampers the pubertal growth spurt. Controlled trials enrolling large series of genetically and clinically characterized PHP-Ia subjects would be necessary to clarify the correct rhGH dosage, the duration of treatment and the eventual combination with GnRH analogs [35].

The evidence of rGHRH in a patient with altered methylation further confirms [26] the possible onset of GH deficiency also in PHP-Ib subgroup of patients and indicates the need of more investigations to assess its prevalence and the correct management in this subtype.

As regards to the AHO signs, among patients with genetic mutations evolutive trends have been evidenced for both SS and OB: the increasing prevalence of SS over the time strengthen the need of a standardized early diagnosis and treatment not only of rGHRH but also of other hormonal derangements that may have an impact on the final stature of these subjects [35]. Conversely, early weight excess, overall OB, supports the hypothesis that an abnormal function of the melanocortin-4 receptors, impairing energy expenditure and insulin sensitivity and inducing early hyperphagia [5, 36], might be responsible for fat accumulation since infancy. The fact that weight excess becomes milder might suggest that other factors influence the energy intake and expenditure, as well as the body composition over the time. The treatment of OB and OW in these patients has been so far limited to suggestions for a balanced nutrition and regular body-exercise; thus, the lack of common standards made the therapeutic approach often inhomogeneous. The inclusion of low-calories and low-fat dietary regimens, the indication of regular physical activity over the time and the development of nutritional education programs as part of the therapeutic approach in these patients and their families will probably help in reducing their metabolic impairment. The feasibility of physical pharmacological treatment to reduce the caloric intake may be evaluated as alternative if dietary approach is unsuccessfully. The possibility to share standardized data searching for insulin resistance in large series would also indicate the correct follow-up and a specific therapeutic approach in those subjects with hormonal derangement. Furthermore, it is important to stress that OB and OW may be related to hormonal impairments but also to a different eating behavior, overall during the evolutive age: standardized endocrinological and psychological follow-up should help clarifying which of them might impact more on fat accumulation.

In this regard, data on mental retardation are still limited; our study further confirms the heterogeneity of the cognitive areas involved in the delay [22] and underlines the need of objective standard tests to verify the most involved areas, also recording the possible evolution of the retardation over the time. Parallel to the evaluation of the straight cognitive impairment, the recent approach to pediatric MR should include standard tests assessing coping skills and adaptive behaviors, which play a central role in how the delay impacts on the daily and social life of the patients [37, 38]. The presence of the sign also in a substantial fraction of patients with imprinting alterations may suggest that even mild reduction in Gsa function, might interfere with the neuronal activity affecting the normal development milestones.

BR is one of the most typical sign of PHP-Ia; its prevalence lower than 100% among subjects with genetic mutations may be explained by their young age (below to the indicated cut-off age of 5 years [39]), and also by the fact that the MPP hasn’t been performed in all subjects, thus potentially underdiagnosing the milder bone alterations. The new prevalence data of 50% among epimutated patients, raises the question if even a slight reduction in Gsα activity might produce specific alterations in hand bone growth: comparative studies are needed to define if it involves the same pattern as in mutated patients.

The only alterations that seem to be specific for genetic mutations are SO, since our series confirms their complete absence in case of locus anomalies [26]. The higher percentage of exon 1 and frameshift mutations among patients showing SO might prove its pathogenetic role as unique one encoding exclusively for Gsα [8]. The young median age of presentation and recent reports indicating possible progression patterns suggests to monitor their evolution in number, size and depth with systematic body-mapping during the follow-up, also in order to differentiate this sign from POH.

## Conclusions

In conclusion, PHP is indeed a very heterogeneous disease, deeply investigated at molecular level in the last decade, for which several clinical aspects have still to be defined in details. Facing its rarity and complexity the ISPED’s Study Group developed the two flow-charts presented in the present paper as diagnostic and therapeutic tool for clinical routine purpose. The proposed pathways should certainly be validated on a larger cohort before their publication as official suggestions or guidelines, but meanwhile, they may be used as standardized schemes in order to obtain uniform clinical data for further studies in the PHP/AHO population.
